# Spontaneous Respiration Using Intravenous Anesthesia and High-Flow Nasal Oxygen (STRIVE-Hi) for Prolonged Rigid Bronchoscopy in a Patient With Functional Single-Lung Physiology

**DOI:** 10.7759/cureus.114537

**Published:** 2026-08-14

**Authors:** Jose A Estrella, Yelidad Llaverias

**Affiliations:** 1 Anesthesiology and Reanimation, Hospital Metropolitano de Santiago, Santiago de los Caballeros, DOM

**Keywords:** airway management, high-flow nasal oxygen, interventional bronchoscopy, spontaneous ventilation, strive-hi

## Abstract

Rigid bronchoscopy in patients with functional single-lung physiology presents a considerable anesthetic challenge because interruption of spontaneous ventilation may rapidly result in severe hypoxemia. Although spontaneous respiration using intravenous anesthesia and high-flow nasal oxygen (STRIVE-Hi) has been described as a technique that facilitates prolonged apneic oxygenation, evidence regarding its application to preserve spontaneous ventilation during prolonged rigid bronchoscopy remains limited. A 55-year-old man with complete obstruction of the left main bronchus secondary to post-intubation tracheobronchial stenosis underwent prolonged rigid bronchoscopy using a modified STRIVE-Hi technique. High-flow nasal oxygen (70 L/min, fraction of inspired oxygen (FiO₂) 1.0) was combined with target-controlled total intravenous anesthesia using propofol (Eleveld model) and remifentanil (Minto model) to maintain spontaneous ventilation throughout the procedure. Bronchoscopy lasted approximately two hours. Four transient desaturation episodes (lowest peripheral oxygen saturation (SpO₂) 83%) occurred exclusively during balloon dilation and resolved after brief procedural pauses without conversion to positive-pressure ventilation or interruption of the intervention. Spontaneous ventilation was preserved from induction to emergence. This case demonstrates the feasibility of a modified STRIVE-Hi approach to facilitate prolonged rigid bronchoscopy while maintaining spontaneous ventilation in a patient with severely limited pulmonary reserve. Individualizing the anesthetic strategy according to respiratory physiology may represent a useful alternative in selected patients undergoing complex airway procedures.

## Introduction

Interventional airway procedures, particularly rigid bronchoscopy, remain among the most challenging scenarios in anesthetic practice because they require a shared airway and are performed through an open ventilatory system in which conventional cuffed ventilation is frequently interrupted or impossible. Consequently, maintaining adequate oxygenation and ventilation can be particularly difficult, especially in patients with significant respiratory compromise. These challenges become particularly pronounced in individuals with severely limited pulmonary reserve, in whom even brief periods of apnea may result in profound hypoxemia.

Spontaneous respiration using intravenous anesthesia and high-flow nasal oxygen (STRIVE-Hi) combines total intravenous anesthesia with high-flow nasal oxygen (HFNO) to optimize oxygenation while preserving spontaneous ventilation during airway surgery. HFNO contributes by delivering a stable inspired oxygen concentration, washing out nasopharyngeal dead space, generating modest positive airway pressure, and reducing the work of breathing [1-3]. Although STRIVE-Hi may also increase tolerance to brief periods of apnea through these physiological effects, its primary objective is to maintain spontaneous ventilation whenever feasible, distinguishing it conceptually from transnasal humidified rapid-insufflation ventilatory exchange (THRIVE), which was specifically developed to prolong safe apneic oxygenation [1-4].

Complete obstruction of a main bronchus may result in functional single-lung physiology, in which only one lung effectively participates in gas exchange, leaving minimal physiological reserve during airway interventions. Reports describing the use of STRIVE-Hi to deliberately preserve spontaneous ventilation during prolonged rigid bronchoscopy in patients with this physiological condition remain scarce [1,4-7]. The present case describes the anesthetic management of a patient with complete left main bronchus obstruction who underwent prolonged rigid bronchoscopy using a modified STRIVE-Hi approach aimed at preserving spontaneous ventilation throughout the procedure.

## Case presentation

A 55-year-old man (weight 120 kg, height 179 cm, body mass index 37.5 kg/m²) with a history of arterial hypertension, class II obesity, and chronic left lung collapse secondary to prior pulmonary tuberculosis was scheduled for prolonged interventional bronchoscopy. Preoperative transthoracic echocardiography demonstrated preserved left ventricular systolic function (left ventricular ejection fraction (LVEF) 70%) without structural abnormalities, and electrocardiography showed sinus rhythm. Cardiovascular risk was considered intermediate based on the Revised Cardiac Risk Index (Lee Criteria); pulmonary risk was high based on the Assess Respiratory Risk in Surgical Patients in Catalonia (ARISCAT) scale. The patient was classified as American Society of Anesthesiologists (ASA) physical status III. Baseline oxygen saturation on room air was 94%.

**Figure 1:**
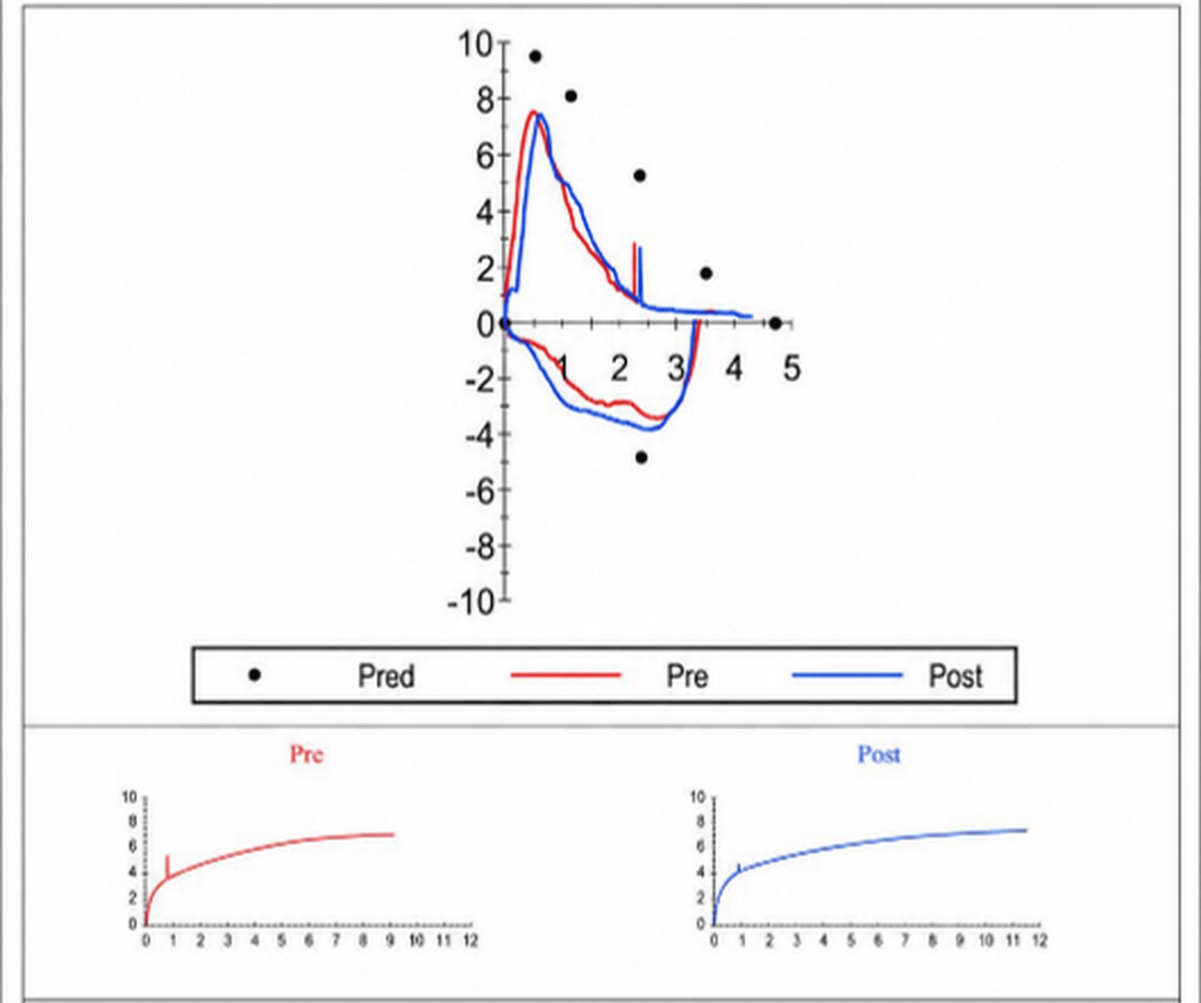
Preoperative spirometry demonstrating a predominantly restrictive ventilatory pattern The flow-volume loop shows reduced expiratory and inspiratory volumes with the preservation of the FEV₁/FVC ratio. Pre-bronchodilator values demonstrated FVC 2.18 L (55% predicted), FEV₁ 2.17 L (67% predicted), and FEV₁/FVC 99.8%, consistent with severe reduction in functional lung volume secondary to chronic left main bronchus obstruction and near-complete collapse of the left lung. FVC: forced vital capacity; FEV₁: forced expiratory volume in one second; FEV₁/FVC: forced expiratory volume in one second-to-forced vital capacity ratio

Preoperative spirometry demonstrated a restrictive ventilatory pattern, with a forced vital capacity (FVC) of 2.18 L (55% predicted), a forced expiratory volume in one second (FEV₁) of 2.17 L (67% predicted), and a preserved forced expiratory volume in one second-to-forced vital capacity ratio (FEV₁/FVC) of 99.8%, consistent with severe loss of functional lung volume rather than diffuse obstructive airway disease. These findings supported the physiological impact of the chronic left main bronchus obstruction and near-complete collapse of the left lung (Figure 1).

**Figure 2:**
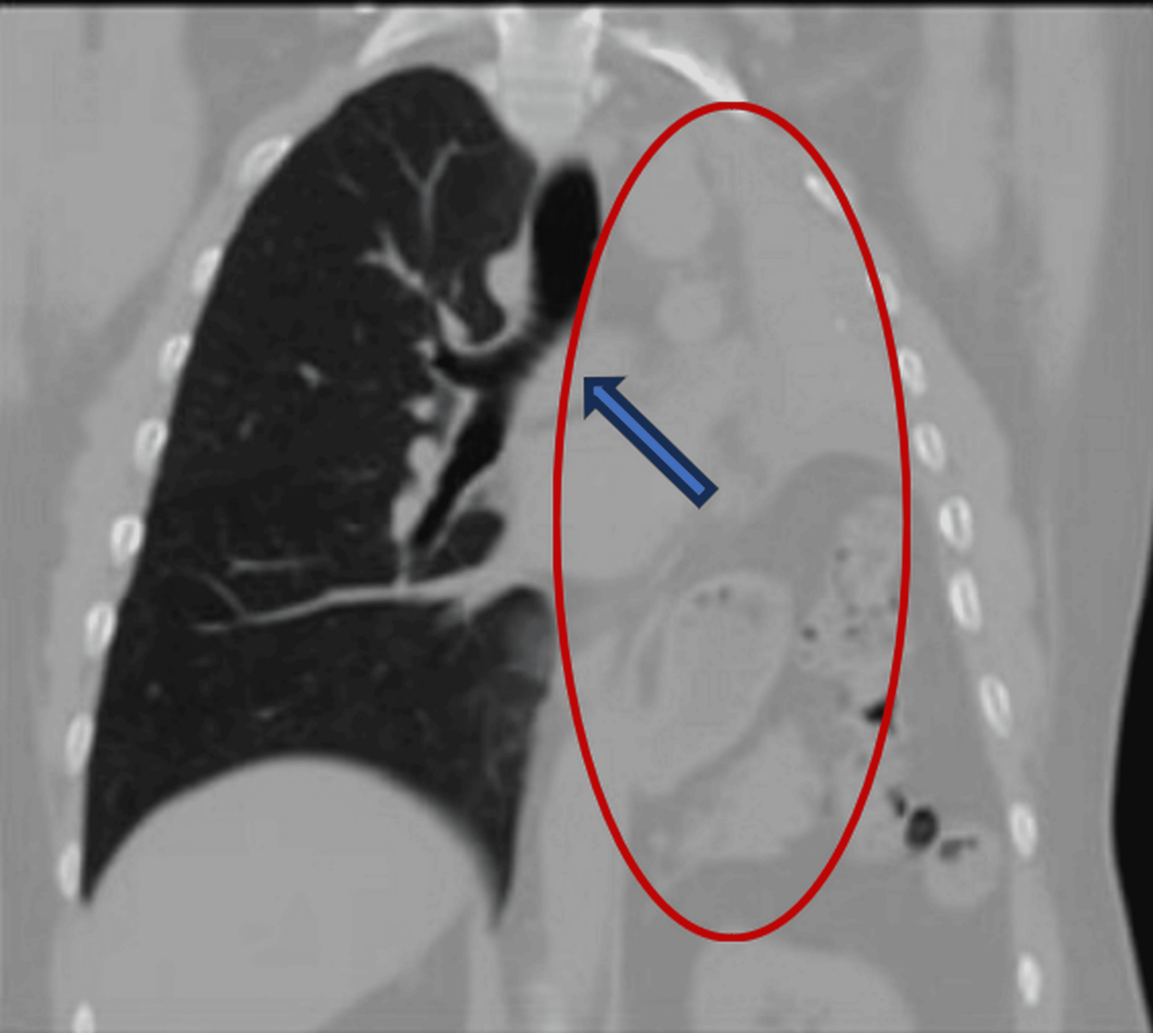
Preoperative coronal chest computed tomography Complete obstruction of the left main bronchus (blue arrow). The red circle outlines the expected anatomical location of the left lung, which is nearly completely collapsed secondary to chronic left main bronchus obstruction, resulting in functional single-lung physiology. The right lung remained fully expanded and served as the patient's only effective gas-exchanging lung during the procedure. These anatomical findings correlated with preoperative spirometry demonstrating severe restrictive ventilatory impairment.

Preoperative chest computed tomography revealed complete obstruction of the left main bronchus with near-total collapse of the left lung, resulting in functional single-lung physiology (Figure 2), with the right lung representing the sole effective gas-exchange surface. These physiological findings correlated closely with the anatomical abnormalities observed on computed tomography, providing objective evidence of markedly reduced pulmonary reserve.

Table 1 summarizes the patient's baseline characteristics and preoperative assessment.

**Table 1:** Baseline patient characteristics and preoperative assessment BMI: body mass index; ASA: American Society of Anesthesiologists physical status; SpO₂: peripheral oxygen saturation; LVEF: left ventricular ejection fraction; ARISCAT: Assess Respiratory Risk in Surgical Patients in Catalonia; RCRI: Revised Cardiac Risk Index; CT: computed tomography; FVC: forced vital capacity; FEV₁: forced expiratory volume in one second; FEV₁/FVC: forced expiratory volume in one second-to-forced vital capacity ratio

Variable	Value
Age	55 years
Sex	Male
BMI	37.5 kg/m²
ASA	III
Baseline SpO₂	94%
LVEF	70%
ARISCAT	High risk
RCRI	Intermediate risk
CT	Complete left main bronchus obstruction
Spirometry	Restrictive pattern
FVC	2.18 L (55%)
FEV₁	2.17 L (67%)
FEV₁/FVC	99.8%

Upon arrival in the operating room, standard monitoring was applied. Baseline vital signs were as follows: blood pressure 140/78 mmHg, heart rate 85 beats/min, and oxygen saturation 94%. HFNO was initiated using an AIRVO™ 2 system (Fisher & Paykel Healthcare, Auckland, New Zealand) at 60 L/min (fraction of inspired oxygen (FiO₂) 1.0, temperature 35°C). During rigid bronchoscopy, an additional oxygen flow of 10 L/min was administered through the bronchoscope side port via the anesthesia circuit, resulting in a total oxygen flow of approximately 70 L/min while preserving spontaneous ventilation.

Total intravenous anesthesia was administered using target-controlled infusions of propofol (initial effect-site concentration 2 µg/mL, Eleveld model) and remifentanil (1.5 ng/mL, Minto model), titrated to maintain spontaneous ventilation while providing adequate procedural conditions. Bispectral index (BIS) values remained between 40 and 60 throughout the procedure after approximately three minutes following induction, avoiding bolus administration to facilitate the preservation of spontaneous ventilation.

During balloon dilation of the obstructed bronchus, the patient maintained spontaneous ventilation with preserved airway reflexes and sympathetic responses. The spontaneous respiratory rate remained between 10 and 14 breaths/minute throughout the procedure. To facilitate balloon dilation, the effect-site concentration of propofol was transiently increased to 3.5 µg/mL, while remifentanil was increased to 3.0 ng/mL. Given the patient's markedly limited pulmonary reserve, remifentanil was subsequently reduced to 2.5 ng/mL to minimize the risk of respiratory depression while maintaining adequate procedural conditions.

The bronchoscopy lasted approximately two hours. Four episodes of oxygen desaturation occurred, with a nadir peripheral oxygen saturation (SpO₂) of 83%, each lasting less than 60 seconds. All events occurred exclusively during balloon dilation in segments with complete obstruction due to dense fibrotic and cicatricial tissue. Episodes resolved promptly following brief procedural pauses and partial withdrawal of the rigid bronchoscope while maintaining HFNO at the same settings, without conversion to positive-pressure ventilation or interruption of the intervention. No clinically significant hemodynamic instability accompanied the desaturation episodes. The principal intraoperative anesthetic and respiratory parameters are summarized in Table 2.

**Table 2:** Summary of intraoperative anesthetic management and respiratory events Ce: effect-site concentration; SpO₂: peripheral oxygen saturation

Procedure phase	Propofol Ce	Remifentanil Ce	SpO₂	Event
Induction	2.0	1.5	94%	Stable
Balloon dilation 1	3.5	3.0	83%	Desaturation
Balloon dilation 2	3.5	3.0	84%	Desaturation
Balloon dilation 3	3.5	3.0	85%	Desaturation
Balloon dilation 4	3.5	3.0	84%	Desaturation
Completion	2.0	2.5	94%	Stable

Continuous sidestream capnography was maintained throughout the procedure. However, quantitative end-tidal carbon dioxide (CO₂) values could not be interpreted reliably because of dilution caused by HFNO. Nevertheless, a continuous capnographic waveform was maintained throughout the procedure without abrupt changes suggestive of apnea or severe hypoventilation. Transcutaneous CO₂ monitoring and arterial blood gas analysis were not available.

## Discussion

STRIVE-Hi has emerged as an effective anesthetic strategy for airway surgery by combining high-flow spontaneous ventilation [1,4]. Most published reports emphasize its ability to prolong safe apneic oxygenation, thereby facilitating complex airway interventions without conventional ventilation [1,4]. In contrast, the present case illustrates a different clinical application. Given the patient's functional single-lung physiology and minimal physiological tolerance for apnea, the primary objective of the modified STRIVE-Hi technique was not to extend apnea but to avoid it whenever possible while maintaining adequate procedural conditions.

Complete obstruction of the left main bronchus resulted in functional single-lung physiology, leaving the patient with markedly limited pulmonary reserve during airway manipulation. This physiological impairment was objectively supported by preoperative spirometry, which demonstrated a restrictive ventilatory pattern (FVC 55% predicted, FEV₁ 67% predicted, preserved FEV₁/FVC ratio). Rather than indicating diffuse airflow obstruction, these findings reflected severe reduction in functional lung volume secondary to chronic collapse of the left lung, confirming that effective gas exchange depended almost entirely on the contralateral lung [8]. Consequently, transient mechanical compromise of the remaining functional airway during balloon dilation was expected to produce rapid oxygen desaturation despite the preservation of spontaneous ventilation. This physiological interpretation is consistent with the intraoperative observation that every desaturation episode occurred during balloon dilation rather than during spontaneous ventilation itself. These episodes resolved promptly after brief procedural pauses, further supporting transient mechanical airway obstruction, rather than failure of the anesthetic strategy, as the predominant mechanism of hypoxemia.

Nevertheless, objective preoperative pulmonary function testing was available and demonstrated severe restrictive ventilatory impairment consistent with the anatomical findings on computed tomography, providing objective support for the markedly limited pulmonary reserve described throughout the case.

HFNO likely contributed to maintaining oxygenation through several complementary mechanisms, including delivery of a stable inspired oxygen concentration, washout of nasopharyngeal dead space, generation of modest positive airway pressure, and reduction of work of breathing [2,3]. Together with preservation of spontaneous ventilation, these physiological effects probably enhanced gas exchange throughout the prolonged intervention despite severely compromised pulmonary reserve.

Published reports have described STRIVE-Hi during airway surgery and rigid bronchoscopy; however, most have focused on prolonged apneic oxygenation [1,4-7]. In contrast, this case highlights adaptation of the technique to a patient in whom apnea was intentionally minimized because of markedly limited pulmonary reserve. Rather than applying a predefined anesthetic strategy, management was tailored to the patient's underlying respiratory physiology while preserving the advantages of HFNO and target-controlled intravenous anesthesia.

This report is limited by the absence of arterial blood gas analysis and continuous CO₂ monitoring during the procedure, precluding the objective assessment of ventilation and CO₂ accumulation. Although spontaneous ventilation was maintained clinically throughout the procedure, the absence of these objective measurements precludes direct quantification of ventilatory adequacy. Therefore, the conclusion that adequate ventilation was preserved should be interpreted with appropriate caution, as it is based on clinical assessment and the available intraoperative monitoring rather than direct physiological measurement. Nevertheless, this experience suggests that adapting STRIVE-Hi to the patient's respiratory physiology may be a feasible strategy when preservation of spontaneous ventilation is considered essential during complex airway interventions.

## Conclusions

This case illustrates the feasibility of a modified STRIVE-Hi approach to facilitate prolonged rigid bronchoscopy while preserving spontaneous ventilation in a patient with objectively documented severe restrictive ventilatory impairment secondary to functional single-lung physiology. Rather than emphasizing prolonged apneic oxygenation, this strategy prioritized maintenance of spontaneous ventilation according to the patient's underlying respiratory physiology. Although broader clinical experience is needed, individualized application of STRIVE-Hi may represent a useful anesthetic option for carefully selected patients undergoing complex airway procedures.
